# Subsistence over symbolism: the role of transnational municipal networks on cities’ climate policy innovation and adoption

**DOI:** 10.1007/s11027-017-9747-y

**Published:** 2017-06-06

**Authors:** Kaveh Rashidi, Anthony Patt

**Affiliations:** Department of Environmental Systems Science, ETH Zurich (Swiss Federal Institute of Technology), Universitätstrasse 16, 8092 Zurich, Switzerland

**Keywords:** Climate mitigation policies, Networks, Policy diffusion, Cities, Climate change

## Abstract

Urban areas account for the majority of global greenhouse gas emissions, and increasingly, it is city governments that are adopting and implementing climate mitigation policies. Many municipal governments have joined two different global city networks that aim to promote climate policy development at the urban scale, and there is qualitative evidence that such networks play an important role in motivating cities to adopt climate policies and helping them to implement them. Our study objective is to test this proposition quantitatively, making use of a global database on cities’ environmental policy adoption, and also taking into account a large number of other factors that could play a role in climate policy adoption. Controlling for these other factors, we find that network membership does make a significant difference in the number of different measures that city governments adopt. We also find that there are significant differences between the two different networks, suggesting that the nature of the services that such networks offer their members can play an important role. Our findings lead to the provision of a set of global mitigation strategies: First of all, joining the city networks can lead to a generation of global strategies which can result into climate mitigation benefits. However, cities are required to select the network which provides proper tailor made policies. Second, in the absence of concrete international commitments at the local level, city networks lay the ground for global governance and enable cities to adopt policies independently and proactively. Third, consideration of co-benefits of climate policies can optimize the development of global strategies.

## Introduction

Cities are the major source of greenhouse gas (GHG) emissions, with urban residents and activities accounting more than 70% of global anthropogenic GHG around the globe (World Bank 2010). Independent of efforts taken by national governments, numerous cities have started to launch their own mitigation policies (CDP 2015). Most of these activities aim at the reduction of GHG in the sectors of energy, transportation, urban land use, and waste (Bulkeley 2010; Xuemei 2007). Prior studies have explored several causes for cities’ adoption of climate policies. Some have focused on the socioeconomic and political status of cities as the major policy adoption drivers, while the others have mentioned that cities’ decisions are influenced by external factors such as the decisions of their national governments.

One factor that may also play a role is the presence of city policy networks. While networks differ from each other, they generally have three defining characteristics. First, membership in the network is voluntary, meaning that cities are free to join or leave. Second, they are non-hierarchical, horizontal, and polycentric; therefore, they are considered to be self-governing. Third, the result of discussions—and consequently, decisions—are normally implemented by the member cities (Kern and Bulkeley 2009). Two networks, the Local Governments for Sustainability (ICLEI, from its previous name International Council for Local Climate Initiatives) and the more recent one known as C40, are the most noticeable transnational networks focusing on climate change mitigation programs, setting the grounds and facilitating the process for knowledge sharing. ICLEI is established in 1990 with the intention to assist local governments in transforming effectively towards a greener economy. It encompasses more than 1500 member cities around the world which represent more than 20% of the world population. ICLEI provides a platform for urban policy makers to exchange recent developments in the adoption of climate policies and the challenges they experienced in planning and implementation phases. ICLEI has offices around the globe and holds annual workshops and conferences in different member cities. C40 is the other large network. It now includes more than 80 cities, representing roughly 10% of the global population. Like ICLEI, the C40 network connects cities to address the issue of climate change through the development and implementation of programs and policies leading to a reduction of GHG emissions. C40 organizes different conferences during the year to help cities learn from each other and provides advice and solutions to the cities. Both networks require their members to pay membership fees on an annual basis. The fees for C40, however, are substantially higher, and there is reason to believe that these higher fees are associated with more-carefully tailored policy analysis. This can be linked to the advisee’s willingness to cooperate and follow up for further advices when he or she has to pay for the service (Patt et al. 2006).

There is evidence that city networks may play an important role in defining and developing climate-policy initiatives at the city level (Fünfgeld 2015). Studies have examined the relationship between city networks and climate policy adoptions; however, these have mainly focused on the question of why cities join climate networks in the first place (Betsill and Bulkeley 2004; Buis 2009; Heinrichs et al. 2013; Kern and Bulkeley 2009; Pitt 2010). Only two studies have addressed the impact that networks have on the policies adopted (Krause 2012; Lee and Koski 2014), and for reasons that we explore in the following section, these leave major question still open, and the evidence remains weak as to whether network membership leads to an outcome of greater climate policy adoption. In this paper, we address this issue empirically. We go beyond prior studies, in that we take into account a wide range of other potential explanatory variables. We also differentiate between memberships in one or multiple networks.

## Background

Policy innovation is the adoption of policies that previously had not existed or are newly adopted by the government. Based on Mohr (1969), one can describe policy innovation as “directly related to the motivation to innovate, inversely related to the strength of obstacles to innovation, and directly related to the resources available for overcoming such obstacles” (p. 114). Several studies have focused on the questions of “What are the drivers of policy innovations in cities?” and “Why are some cities more innovative than the others?” Walker (1969), in studying the diffusion of innovation and policy making among the American states, suggested that the likelihood of one state adopting a new policy is higher when others have already implemented it. In other words, cities tend to emulate policies that have already been tested elsewhere. Berry and Berry (1990) emphasized that the process of policy innovation in cities is influenced by not only internal factors such as socioeconomic developments, geographic characteristics, and political ideologies but also external factors that lead to the diffusion of ideas.

Internal determinants of policy innovation have been shown to play an important role. Cities’ policy adoption can be linked to factors such as demographics, socioeconomic development, geography, and political ideology (Canon and Baum 1981; Gray 1973; Walker 1969). Several studies have drawn on this theory to examine the particular case of policies for climate adaptation and mitigation. Ryan (2015) listed three barriers to the adoption of climate policies by cities: lack of sufficient funding, adequate human resources, and information management; those cities that overcome these barriers are more likely to adopt more climate policies. The relationship between income and policy adoptions has been assessed by Collier (1997) and Mathy (2007); both observed positive correlations between income and the number of climate policies adopted. Another socioeconomic factor is the population of the cities. Betsill (2001) and Lee (2013) noted that more-populous cities have more resources to dedicate to the promotion of climate policies. Moreover, cities with more population are endowed with increased civic capacities and tend to push for more climate policies (Krause 2012; Lee and Koski 2014). The influence of citizens’ awareness of their governments is another factor that shapes the decisions of policy makers (Pierson 1993). One line of research has linked public awareness to the employment of women at various levels of occupations. Higher levels of female employment are an indicator of a society’s open mindedness and a government’s positive attitudes towards human rights and surrounding environments. Torgler and Garcia-Valiñas (2007) found that women are generally more worried about climate change and related environmental problems.

Geographic and local environmental factors also play a role, with a propensity towards natural hazards being a strong motivator of policy. Panayotou (1997) observed that governments react to disasters that they have experienced directly rather than disasters observed from afar. During recent years, the number of climate change-related hazards, such as hurricanes and coastal storms, has increased significantly, and when combined with projections of sea-level rise, one could expect that coastal cities would adopt more climate policies (Lee 2013; Nicholls et al. 2008) There are several studies that emphasize the importance of co-development benefits for climate policy (Bulkeley and Betsill 2005b; Krause 2011). Reduced fossil fuel consumption and air pollution are the most apparent local outcomes of many climate policies, with measureable improvements in public health and air visibility. Lowering fossil fuel consumption also reduces the dependency on importing energy, with benefits on balance of payments. One could expect that communities that are heavily dependent on the consumption of fossil fuels would be more likely to adopt certain climate policies, such as those that promote the installation of insulation systems in buildings, reducing energy consumption and the associated dependency on imported fossil energy sources. A large number of cities suffer from air pollution, and governments have adopted various programs and initiatives to limit aerosol particulates (Betsill 2001; Lindseth 2004). Ryan (2015) found that for some cities, the co-benefits of climate policies are even more important as a motivating force than the mitigation of GHG emissions: co-benefits are simply more tangible, something that citizens can feel, compared to global benefits that come from reducing greenhouse gas emissions. These environmental factors can interact with policy makers’ ideological beliefs: the literature shows that governments that have implemented other environmental policies, addressing local concerns, tend to be more enthusiastic to adopt climate policies (Betsill 2001; Zahran et al. 2008a, b).

In addition to internal factors leading to policy adoption, the literature also highlights the importance of external factors (Berry and Berry 1990). These have to do with the institutional and governance context within which the city operates and the mechanism by which external factors operate can be classified as either vertical or horizontal diffusion. In vertical diffusion, policies are directed by the national governments to the local governments. These national decisions may thus shape the climate policies that are going to be adopted by municipal governments (Daley and Garand 2005). There is anecdotal evidence suggesting that cities in countries that had accepted the United Nations Framework Convention on Climate Change Kyoto Protocol earlier adopted more climate programs than those that joined later. National governments’ influence can also vary depending on the governing structure. In countries with a centralized (top-down) political system, the implementation of nationally legislated environmental and climate change policies seems to be more effective and quicker (Kimber 1995).

The idea of horizontal diffusion suggests that the policies can be transferred from one city to another. Rogers (2010) defined such horizontal diffusion as “the process by which an innovation is communicated through certain channels over time among the member of a social system” (p. 162). Berry and Berry (1990) emphasized that learning from the experience of the others and competition among cities are the primary drivers of horizontal diffusion. Hence, if one city implements a new policy and finds the policy to be successful and popular, there is a reasonable chance that the others that are informed of that innovation will adopt it as well.

Two avenues for horizontal diffusion are attending international conferences and being a member of transnational networks. In fact, the two can be related, since transnational networks are a potentially important forum for local leaders to present their ideas, experiences, and achievements, as well as to learn from others’ activities and expertise (Betsill and Bulkeley 2004; Meseguer 2005). Municipalities are now more connected than ever before, with connections made through networks, and provide opportunities for “intermunicipal dialog,” which results in global influences (Toly 2008). Local government associations can encourage professionalism for the member cities, especially those located in developing economies. Networks can play a role in “agenda settings” (Buis 2009). Busch (2015) believed that their functionalities are even more as they can be “consultant,” “advocate,” “commitment brokers,” and “platforms” and thereby might result into different impacts on their members. Focusing on the Cities for Climate Protection (CCP) program, ICLEI (Betsill and Bulkeley 2004; Bulkeley et al. 2003) concluded that the networks lay the groundwork for information and knowledge sharing. The networks also provide situations in which the member cities can compete with each other (Kern and Bulkeley 2009) and find the right partners to with which they could team up to attract external financial supports (Bulkeley et al. 2003).

But networks can also be helpful in capacity building and rule setting (Andonova et al. 2009; Bulkeley et al. 2003), whereby the network operates as more than simply a venue for information exchange, but actually serves as in institutional locus of expertise and procedural guidance. They can provide benchmarking, recognition for cities, and some form of certification for the members that have achieved certain climate-policy goals (Kern and Bulkeley 2009).

There are increasing numbers of cities joining these climate mitigation city networks. Network studies have extensively addressed the questions of why cities join the networks (Betsill and Bulkeley 2004; Pitt 2010), what recognition benefits cities can expect (Buis 2009; Kern and Bulkeley 2009), and how memberships can provide visibility to leverage international funding (Betsill and Bulkeley 2004; Heinrichs et al. 2013). Moreover, the literature on cities’ climate policies and networks has mainly been qualitative in nature (Bontenbal 2013; Bulkeley 2005; de Jong et al. 2015; Schroeder et al. 2013; Toly 2008; Zeppel 2013), and studies that provide quantitative assessments are limited to a specific geographical boundary and do not provide generalized solutions (Busch 2016; Davies 2005; Kern and Bulkeley 2009; Krause 2012; Matisoff 2008; Pitt 2010). City networks, despite their increasing importance, are “… still relatively poorly understood with regards to their influence and impact on environmental outcome in local areas” (Fünfgeld 2015) and “net effects of cities participation in transnational networks” are not measured (Fünfgeld 2015).

Two studies have gone the furthest in identifying the relationship between city network membership and climate policy adoption. Assessing the impact of ICLEI and the Mayors Climate Protection Agreement (MCPA) network membership in United States (US) cities, Krause (2012) applied ordinary least square (OLS) and propensity matching scores to answer the question of whether network membership had an influence on climate policy adoption. Krause found that it did, but left several issues unexamined. First, Krause studied only diffusion among American cities, raising the question of whether similar effects could be seen internationally, in particular in developing countries. Second, Krause studied a limited set of potential climate policies, leaving the question open of whether policies more tangentially related to mitigation would show the same effects. Third and related, Krause did not examine the question whether co-benefits to such policies could be an important driver. Lee and Koski (2014) reached a similar finding, doing so in an international context, but their methods raise some questions. They examined cities that were members of the C40 network and then looked at the relationship between additional membership in the ICLEI network and policy adoption. In this sense, their sample was already biased towards those cities that had already joined at least one network. Like Krause (2012), they also failed to take into account co-benefits as a potential driver. In light of these limitations, Fünfgeld (2015), writing a review paper of the field, highlighted a continued gap in the evidence for network membership effects.

In our study, using a database of more than 127 cities, we address the following questions. First, can we observe a global impact of city network membership, in terms of the number climate policies that cities adopt? Second, and more nuanced, is there any difference between the city climate networks of C40 and ICLEI in terms of their impact on climate-policy adoptions? In studying this difference, we can differentiate between the horizontal transfers that take place when the network serves primarily as a forum for interaction, compared to when the network’s own institutions attempt to add additional value.

## Methods

We have compiled data from various existing sources. The primary data sources are the reports submitted by cities to the CDP (formerly known as Carbon Disclosure Project) database (CDP 2015), which hosts a global database of cities, companies, and governments where environmental impact reports, actions on climate policies, and carbon footprints are reported to the CDP based on a self-reporting mechanism. The database of CDP is widely used in research and policy making arenas. For variables related to a city’s dynamics, we use secondary data sources extracted from public sources and city municipality websites. We also introduce several explanatory variables controlling for environmental background of the cities to reduce the potential bias which may be caused by the voluntary nature of reporting the policy adoptions. Tables 1 and 2 present a full list of variables along with their sources. The dataset covers 127 cities around the world and includes 1651 data points in total, which is large enough to run statistical estimations.

**Table 1:** Policies included in the dependent variables

Category	Policy
Energy demand in buildings	Transmission and distribution loss reduction
	Building codes and standards
	Smart grid implementation
	Low or zero carbon energy supply generation
	Combined heat and power
	Building performance rating and reporting
	Renewable onsite energy generation
	Financing mechanism for retrofit
	Clean energy procurement strategies
Finance	Carbon finance capacity building
	ESCO financing
	Carbon finance/markets
	Clean technology funds
Outdoor lighting	LED, CFL, and other luminaire technologies
	Solar powered lights
	Smart lighting
Public procurement	Encourage low carbon products
Transport	Improve fuel economy and reduce CO_2_ from motorized vehicles
	Improve the accessibility to public transit systems
	Infrastructure for non-motorized transport
	Improve bus transit times
	Improve the efficiency of freight systems and transport demand management
	Improve fuel economy and reduce CO_2_ from bus and/or light rail operations
Urban land use	Compact cities
	Limiting urban sprawl
	Urban agriculture
	Transit oriented development
	Eco-district development strategy
Waste	Waste to energy
	Methane recovery for reuse
	Improve the efficiency of waste collection
	Landfill gas capture
	Integrated waste management
	Wastewater to energy initiatives
Education	Climate change-focused curriculum

**Table 2:** List of independent variables

Variable	Definition	Source
Socioeconomic and geographic		
Income	Logarithmic values of GDP per capita for the cities	City municipalities, National Bureau of Statistics
Population	Logarithmic values of population of cities	City municipalities, National Bureau of Statistics
Employment	Percentage of female employment with secondary education	World Bank
Coastal location	Binary variable, if city is coastal, it takes 1 otherwise 0	Authors’ desk-based research
Co-development benefits		
Fossil fuel consumption	Percentage of share of fossil fuel use to total energy consumption	World Bank
Air pollution	Normalized values of air pollutions in cities	World Bank
Policy variables		
Environmental policies	Normalized values of non-climate environmental policies	CDP
Target	Binary variable, if city has GHG mitigation target, it takes 1 otherwise 0	Carbonn and C40
GHG (millions)	Total amount of GHG in millions reported by cities	ICLEI, C40, CDP, and cities municipalities
Vertical diffusion		
National policies	Years passed since the country accepted Kyoto protocol	UNFCCC
Governing structure	Binary variable, if political system is top-down, it takes 1 otherwise (federal) 0	McGill (2016)

In order to assess the impact of city network membership on the adoption of climate policies, the following regression model is proposed

1 $$ Y={\beta}_0+\sum_{j=1}^k{\beta}_j{C}_j++\alpha E+\delta G+\gamma H+\varepsilon $$

In Eq. (1), *Y* denotes the total number of adopted policies by the city, *β*_0_ indicates the constant variable, *C*_*j*_ represents the control variables for the drivers of policy adoption while *β*_*j*_ indicates the coefficients on each of those drivers. Two variables of *E* and *G* control for vertical diffusion of policies. The *α* reflects the coefficient of control variable *E* for government’s commitment towards climate change, *δ* is the coefficient for the governing structure *G*, *γ* is the coefficient for main variable *H*, *ε* is the error term, and *k* reflects the total number of variables in bundle of control variables.

## Dependent variable

The policy count variable *Y* measures the total number of climate policies implemented by each city from 2010 to 2013. It encompasses policies from the following sectors: energy demand in buildings, finance, outdoor lighting, public procurement, transport, urban and land use, and waste and education. In total, a city can gain a maximum of 35 points for climate policies adopted. The dependent variable considers the algebraic sum of all the climate policies. Notably, only the policies that directly impact the GHG emissions have been selected. Table 1 lists these policies and the categories to which they belong.

## Independent variables

The independent variables *C*_*j*_ and *H* from Eq. 1 are listed in Table 2. In addition to the main variable counting network membership, we include a theory-driven set of control variables, also listed in Table 2. Going beyond previous studies, we include a set of variables capturing co-benefits of climate policy and a variable capturing the intrinsic propensity of a city to engage in environmental policy-making more generally. This latter factor makes use of the number of environmental policies in place other than those with a clear impact on climate mitigation or adaptation. Examples include the establishment of green spaces for recreation.

### Socioeconomic and geographic variables

We have gathered GDP per capita from global databases and city municipalities for the income variable. We adjusted the values to 2009 US dollars, and we used the GDP deflator from the World Bank Database. We used logarithmic values of income to have a better distribution. We also issued logarithmic values of population for cities’ inhabitants. The hypothesis is that cities with larger populations will adopt more environmental and climate policies. Krause (2012) and Lee and Koski (2014) used a similar approach. The data have been gathered from the city databases and national statistics bureaus.

National level variable values are extracted from the World Bank Database. The percentage of female employment with secondary education has been used as the proxy for educational level on climate change issues. Numerous studies have investigated the relationship between women and environmental concerns. For instance, Torgler and Garcia-Valiñas (2007) mention that in general, women demonstrate a greater level of concern towards environmental issues. Also, a larger level of women’s working age population correponds to “a stronger preference for enivornemntal quality” (Zhao et al. 2013). We would like to extend this further by examining relationship between females’ educational background and adoption of climate policies. The hypothesis is that a society which has more educated women at different employment levels should adopt more climate policies.

A dichotomous variable for coastal location is used, taking the value of 1 if the city is coastal and 0 otherwise. Coastal areas are the first to be affected by climate change. As Nicholls et al. (2008) highlighted, coastal cities have either experienced the devastations caused by the climate change (in the form of coastal storms) or they are expecting it. In both cases, adaptation and mitigation policies would be required: one for the short-term preparedness in addressing local concerns in case of disaster (placing appropriate adaptation measures); the other is the policies with lasting impacts that could mitigate, eliminate, and avoid the occurrence of such disasters (applying climate mitigation policies) (Lee 2013; Nicholls et al. 2008). We, therefore, hypothesize that as the coastal cities are probably more exposed to the immediate impacts of climate change, they are more informed about the negative impacts and take more initiatives and policies to deal with it through not only implementing adaptation measures but also looking for ways to mitigate the likelihood of occurrence.

### Co-development benefits

We have considered two variables to capture the co-development reasons that cities adopt climate policies. Fossil fuel use measures the share of fossil use against total energy consumption. We use the normalized values of cities’ air pollution from the World Bank Database.

### Policy variables

Environmental policies unrelated to climate serves as a proxy for the local government’s political willingness to adopt climate policies. We include this variable to test the hypothesis that the higher number of policies adopted by a city to address environmental issues other than climate change (e.g., local air pollution), the more climate change mitigation policies are likely to be adopted as well. In other words, governments with greener attitudes adopt more climate policies (Betsill 2000; Bulkeley and Betsill 2005a). We extracted the values from the CDP database and normalized between 0 and 1. GHG target setting is measured as a binary variable to test the local government’s seriousness in tackling climate concerns. In the wake of climate change discussions, many cities have expressed their willingness to tackle GHG emissions by setting targets for reductions by 2020, 2025, or 2030. Target setting is an indicator of taking concrete steps in planning, designing, and implementing climate actions and in removing barriers such as a lack of human and financial resources (Holgate 2007). The other control variable is the logarithmic values of greenhouse gases per tons of CO_2_ emitted by cities. The assumption is that cities with a higher amount of GHG emissions have more incentive to adopt climate policies. The data has primarily been gathered from the CDP, Carbonn (a platform on which cities enter their GHG inventory data), and city municipalities. For the few cities without GHG accounting systems, national values—proportionally adjusted to the population—are used.

The vertical diffusion element consists of two variables. First is the national policy variable which measures the years elapsed since the acceptance of Kyoto protocol (incorporated as *E* in Equation 1). This variable is in fact a policy variable that measures the influence of national governments on cities’ climate policy adoptions (commitments at the national level) as has been used similarly by Lee and Koski (2014). The list of countries with the years of acceptances has been extracted from the United Nations Framework Convention on Climate Change (UNFCCC) website. Similar to the work of (Lee 2013), we also introduce a variable to control for governing structure (indicated as *G* in Eq. 1) which measures the impact of governing system on adoption of climate polices at cities and sub-sovereign states. It is a binary variable and takes the value of 1 if the structure is top-down and 0 if it is otherwise, i.e., bottom-up (federal). This could matter, in the case of a governance system, whereby the central government has not only made international climate commitments but has also mandated city governments to adopt particular policies. These data have been collected from the list provided by McGill (2016).

The main variable *H*, horizontal diffusion of policy through network membership, is a group of three dummy variables: one for membership in each of ICLEI and C40 and one interactive being membership in both networks. We have chosen these two networks based primarily on their global coverage, and the fact that they potentially contribute to a wide spectrum of climate policy adoption. Hence, we do not include data on membership in the Climate Alliance or the Covenant of Mayors, both of which are limited to Europe. We also do not examine membership in the Energy Cities network, which focuses on policies in the energy sector. With respect to the ICLEI network, we do not make use of data on cities’ membership in a particular ICLEI program, the Cities for Climate Protection (CCP). The CCP program is associated with cities adopting a particular set of climate policies, and membership is restricted to those cities that have done so. By contrast, participation in the wider ICLEI network may have an effect on cities’ policy adoption, but is not contingent on it. It is also worth noting that ICLEI has now rebranded the CCP and integrated into other agendas (van Staden 2017; Yi et al. 2017).

## Results

**Fig. 1:**
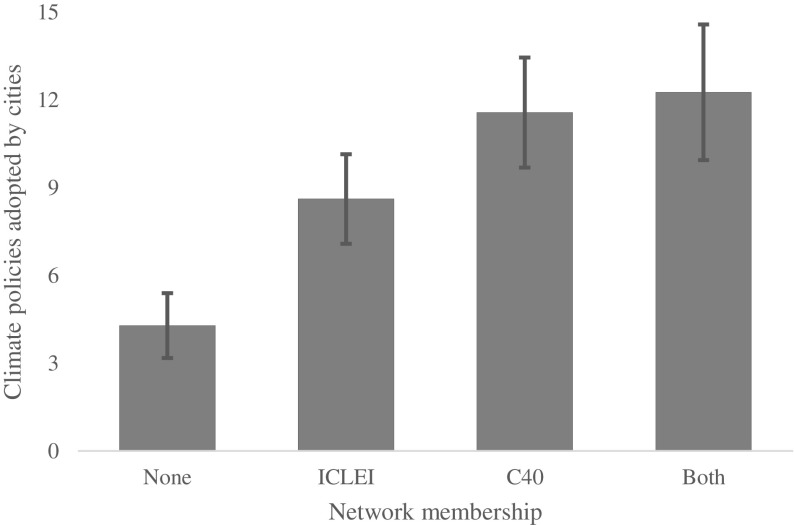
The diagram shows the mean within cities policy adoption as a function of city networks. *Error bars* represent ±1.96 standard errors, the 95% confidence interval. Cities that are a member of both of the networks have adopted more climate policies than those that are members in either or none of the networks

Figure 1 provides a first approximation of the effect of network members. It shows the mean number of climate policies adopted, differentiated by cities’ membership in climate networks. Cities belonging only to ICLEI adopt more policies on average than those belonging to no networks, while cities belonging to C40, either instead of or in addition to ICLEI, adopt more policies than those belonging only to ICLEI. There is no statistical difference between those cities belonging only to C40 and those belonging to both networks, whereas the differences between other means are significant at the 95% confidence interval.

While indicative of an overall trend, Fig. 1 does not reveal whether other factors, potentially correlating with network membership, could be explaining the observed differences in policy adoption. We used three regression models in order to do so, each of which could be considered appropriate for our data structure: ordinary least squared (OLS), Poisson, and negative binomial (NB). In all cases, the results from Poisson and NB models agree qualitatively, while OLS results are in some contradictory with respect to the effects of control variables. However, OLS is somewhat problematic because the residuals are not normally distributed (*p* < .001). Moreover, overdispersion tests show that the null hypothesis of having no overdispersion is rejected, as *p* < .001, suggesting that of the three models, the NB provides the most efficient and robust estimates. We present all three models, but draw our qualitative conclusion from the NB results. Table 3 shows the results based on three estimation techniques.

**Table 3:** The results for the OLS methods and negative binomial methods (*N* = 127)

Variables	OLS			Poisson			NB		
Socioeconomic and geographic									
Income	3.253	*	(1.235)	1.990	***	(1.181)	2.015	***	(1.218)
Population	−1.031		(0.661)	0.937		(1.084)	0.939		(1.103)
Employment	−0.075	***	(0.022)	0.989	***	(1.003)	0.987	***	(1.003)
Coastal location	0.728		(0.607)	0.985		(1.074)	0.971		(1.092)
Co-development benefits									
Fossil fuel consumption	−0.045	*	(0.024)	0.997	**	(1.002)	0.993	**	(1.003)
Air pollution	5.142	*	(2.975)	3.130	**	(1.495)	3.026	*	(1.618)
Policy variables									
Environmental policies	12.870	***	(1.295)	3.397	***	(1.137)	3.731	***	(1.179)
Target	1.310		(0.744)	1.221	**	(1.098)	1.214	*	(1.117)
GHG	0.000	***	(0.000)	1.000	**	(1.000)	1.000	**	(1.000)
Vertical diffusion									
National policies	0.018		(0.112)	0.984		(1.013)	0.983		(1.016)
Governing structure	0.452		(0.799)	1.005		(1.092)	1.035		(1.116)
Horizontal diffusion									
Networks (C40)	1.893	*	(1.071)	1.440	***	(1.127)	1.392	**	(1.159)
Networks (ICLEI)	0.677		(0.786)	1.283	**	(1.113)	1.274	*	(1.134)
Networks (both)	3.594	***	(1.041)	1.654	***	(1.127)	1.691	***	(1.157)
Intercept	1.959		(7.756)	0.826		(2.650)	0.812		(3.219)
*R* ^2^	0.704			0.697			0.697		

Number in brackets indicate standard errors

*Statistical significance at 90% confidence level; **95% level; ***99% level

What these results allow us to see, as cannot been seen in Fig. 1, is the influence of the network membership variables when taking the control variables into account. In contrast to Fig. 1, it shows that ICLEI membership alone makes only a marginally significant impact on average numbers of policies adopted. Membership in the C40 or both networks makes a larger and clearly significant impact. On average, cities belonging to both networks adopt 1.69 more policies than those cities that are not members. This effect is roughly the same as is accounted for by a doubling of per capita income, close to half of the potential variance on the adoption of other environmental policies, and over half of the potential variance on air pollution. Vertical diffusion was found to have no significant impact on the cities’ decisions in adopting climate policies.

In terms of these other variables, income is significant at the 99% level, supporting the hypothesis that richer cities have a larger budget for climate change policies. Female employment with higher education is also significant at 99%. No significant correlations were found for population or coastal locations. Both of the co-development benefits variables are statistically significant. Cities with more dependency on fossil fuel adopt more climate policies. A 1% increase into the dependency on fossil fuel consumption increases climate policies adopted by 0.993. Air pollution also has a positive significant relation with the city’s climate policy adoption.

Policy variables also show significant positive correlations with the adoption of climate policies. Cities that have implemented more environmental policies also adopt more climate policies, as do cities that have set GHG reduction targets. Cities with higher GHG emissions are more likely to adopt climate policies.

**Fig. 2:**
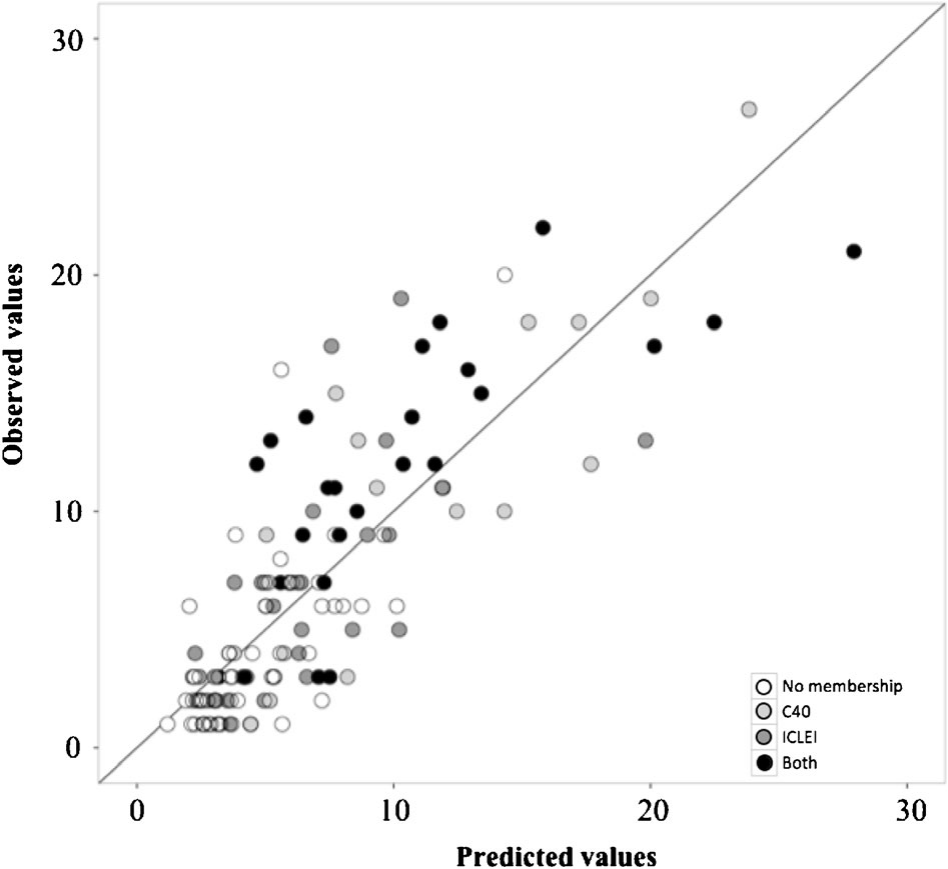
Plot of observed values of climate mitigation policies, compared to those predicted using the estimated model, and considering all factors other than network membership. The *shaded circles* represent the network membership of cities, and *points* above the 45° line represent the outperformers. As is shown in graph, the cities that belong to either or both of the networks are generally located *above the line*

Figure 2 illustrates graphically the results with respect to network membership. It plots predicted values for climate policy adoption based on the control variables, compared to observed values. What can be observed is that with few exceptions, cities that are members of both networks lie above the 45° line, indicating that observed policy adoption exceeds that which would be adopted based on other factors.

## Discussion and conclusion

The results show that joining international networks correlates with an increase in the adoption of climate policies. The results also show that cities in both networks adopt more climate policies than those in only one. Cities that are a member of the C40 network are more likely to adopt climate policies than those that are a member of ICLEI. One can speculate on several reasons, including the fact that C40, with fewer members, can offer a higher level of support to each member. C40 with 90 employees has to cope with 80 members (in theory above one person per city) while ICLEI with 200 employees works with 1500 member cities (less than 0.13 person per city). Another potential reason could be the higher cost of entry, consistent with other studies that show that policy advice is more frequently followed when the advisee has to pay for it (Patt et al. 2006). Moreover, networks with different profiles can play roles of a consultant, an advocate, a “match maker” or a commitment broker (Busch 2015), and one network can be strong in one or all of these features. This may lead to variations in the quality of services delivered, and potentially justify the differences in the likelihood of adopted policies by the member cities. Our results indicate that membership plays a role even when considering other important, and previously unexamined, driving factors.

Most of the results with respect to the various control variables are consistent with the existing literature. The results suggest that the level of air pollution impacts the adoption of climate policies by cities. Cities are interested in the adoptions of climate policies that have public health and economic development co-benefits (Bulkeley and Betsill 2005a). Reducing air pollutants and increasing visibility are tangible co-benefits for several mitigation programs. The policy implication for development and research institutes is that when suggesting climate policies for cities, they should also place sufficient emphasis on the importance of co-benefits. The other co-benefits of climate policies are the reduction of fossil fuel consumption as the result of energy efficient activities. Apart from the few regions that produce their own fossil fuel, the rest are heavily dependent on the fossil fuel imported from somewhere else. Therefore, as was expected, the findings show that an increase in the share of fossil fuel consumption increases the adoption of climate policies. Among the socioeconomic factors, income and female’s employment influence the policy makers’ decisions in the adoption of the climate policies. The findings show that cities with higher incomes adopt more climate policies. Two possible interpretations can be made from this finding. As opposed to cities in developing economies, cities with higher income have already dedicated resources for climate mitigation programs in their strategic development plans in consideration of the potential benefits and budget requirements. The results are consistent with previous literature that reports the importance of financial sources for the development of mitigation policies (Collier 1997; Mathy 2007) and the promotion of innovation in the cities (Mohr 1969), as well as the finding that successful implementation of policy innovations require larger administrative staffs with the knowledge of mitigation policies that can collaborate with contractors (Collier 1997; Mathy 2007). This is the case for wealthier cities where more individuals for mitigation programs can be allocated. Finally, we observe that higher rates of employment of females with higher education have a positive relationship with the adoption of climate policies. The positive correlation between higher education of inhabitants and the adoption of climate policies has already been found (Zahran et al. 2008a, b). Women are more concerned about environmental and climate change problems (Torgler and Garcia-Valiñas 2007) and therefore have different levels of involvement in occupations and local communities that help promote climate change concerns.

One previous study has found a negative correlation between GHG emissions and the adoption of climate policies (Zahran et al. 2008a, b). We find the opposite. The underlying reason could be that cities have become more concerned about climate change and their GHG footprint. In several cities, such as Shanghai and Tokyo, there exist city-level trading schemes that seek to adopt climate policies in response to their massive GHG emissions. We also find that cities that have already implemented environmental initiatives are also more willing to adopt climate policies. This is linked to the capacities that are built in these regions. Politically liberal and educated communities with a successful history of environmental activities react properly to the negative impacts of climate change and support the adoption of mitigation programs (Zahran et al. 2008a, b). Our results also show that cities that have set fixed targets for the reduction of GHG emissions (compared to the base year’s scenarios) adopt more climate polices. We have also found that vertical diffusion has no impact on the adoption of climate policies. The policy implication is that, for cities without concrete national commitment, the decisions regarding the adoption of climate policies can be made independently. For instance, in the absence of solid national commitment and law enforcement, various US cities have been actively engaging in climate change dialogs and adopting related policies. This can be linked to the fact that financial and fiscal autonomy can potentially bring about empowerment to cities so they can adopt more efficient policies and allocate financial sources effectively (Lee 2013; UN HABITAT 2009). On the other hand, even in areas where commitments exist, cities can be more ambitious in adopting policies. This has been highlighted in the conference of parties (COP) 21 agreement (COP 21 2015) and conforms with current practical cases. For example, in Japan, with a persistent national commitment, some cities were able to reach the mitigation targets faster than what was instructed to them. The very good example is the cap-and-trade system initiated by the city of Tokyo (Nishida and Hua 2011) that has led to a significant amount of GHG emission reduction of 25% only in 5 years, much earlier than the initial plans (City of Tokyo 2016).

One potential limitation bias in our results could stem from the voluntary nature of cities’ contribution to the CDP database. It is possible that cities less interested in enacting climate policies, and not joining a network, would not appear in our sample of cities. If such a selection bias exists, however, the result would be to decrease the observed effect of network membership. Hence, we can say that our results are, in this respect, conservative. It is possible that the effects of network membership are greater than those that we have observed. Another potential bias in such empirical studies is that resulting from unobserved variables. One can surmise, for example, that an unobserved variable accounts for network membership and climate policy adoption, meaning that the relationship observed between the two is not causal.

We believe that we have gone beyond previous studies by including a wide range of such potential variables. In particular, we have included the variable for the adoption of environmental but non-climate policies, which we believe can be an indicator for cities’ overall commitment to the environment. What we find is that including these variables makes the observed correlation between ICLEI membership and climate policy adoption appears to be insignificant, whereas without the inclusion of these variables, it had appeared to be significant. This stands in contrast to the results for C40 membership, membership in which persists as significant even with the additional variables added. This suggests two things. First, it suggests that the effect observed from C40 may not be as a result of a previously unobserved variable. Second, and more importantly, it suggests the existence of causality between C40 membership and policy adoption.

It is important to note that we have not measured an adopted policies’ impact. In other words, the policies adopted through joining city networks might have larger mitigation impacts for some cities but have lesser impacts in others. Examining this issue, Pitt (2010) and Zhao et al. (2013) apply similar methods and arguments in their estimations of environmental policies and their impact on governments’ climate actions. We too would likely have been able to observe whether cities that adopt more policies actually make more progress in terms of emissions reduction or the diffusion of low carbon technologies. The fact is, however, that it is too soon to be able to measure such effects. The data for climate policy adoption only commence in 2012, and there are no data yet available to be able to estimate the effectiveness of these measures. This can be viewed as an important area for future research.

City networks are increasingly receiving attention from cities around the world. In this paper, we have attempted to analyze the impact of membership in these two city networks on the adoption of climate policies at cities. Using a large global dataset of cities and controlling for economic, political, environmental, and other policy variables, we have found that joining city networks correlates with the adoption of more climate policies by cities. We also found that across the cities of our study, cities that are a member of C40 on average adopt more climate policies than those of ICLEI. Joining both of the networks increases the likelihood of more policies adopted; however, the impact is not significantly higher than joining either of the networks. Consistent with the previous literature, we have found that other socioeconomic, demographic, policy, and environmental variables also increase the policy adoption likelihood. Our results contribute to the provision of a set of global mitigation strategies through evaluation of the role of international city networks in shaping policy decisions. City networks can contribute to the generation of global strategies, which in turn can translate into climate mitigation benefits. Our results suggest that cities should decide to choose networks that provide sets of tailor-made policies that meet their actual requirements.
